# Above- and belowground linkages during extreme moisture excess: leveraging knowledge from natural ecosystems to better understand implications for row-crop agroecosystems

**DOI:** 10.1093/jxb/erad045

**Published:** 2023-02-04

**Authors:** Christine D Sprunger, Alex Lindsey, Ainsley Lightcap

**Affiliations:** W.K. Kellogg Biological Station, Michigan State University, MI, USA; Department of Plant, Soil, and Microbial Sciences, Michigan State University, MI, USA; School of Environment and Natural Resources, The Ohio State University, OH, USA; Department of Horticulture and Crop Science, The Ohio State University, OH, USA; School of Environment and Natural Resources, The Ohio State University, OH, USA; University of Birmingham, UK

**Keywords:** Above- and belowground linkages, biodiversity, climate change, crop production, flooding, resilience, trophic interactions

## Abstract

Above- and belowground linkages are responsible for some of the most important ecosystem processes in unmanaged terrestrial systems including net primary production, decomposition, and carbon sequestration. Global change biology is currently altering above- and belowground interactions, reducing ecosystem services provided by natural systems. Less is known regarding how above- and belowground linkages impact climate resilience, especially in intentionally managed cropping systems. Waterlogged or flooded conditions will continue to increase across the Midwestern USA due to climate change. The objective of this paper is to explore what is currently known regarding above- and belowground linkages and how they impact biological, biochemical, and physiological processes in systems experiencing waterlogged conditions. We also identify key above- and belowground processes that are critical for climate resilience in Midwestern cropping systems by exploring various interactions that occur within unmanaged landscapes. Above- and belowground interactions that support plant growth and development, foster multi-trophic-level interactions, and stimulate balanced nutrient cycling are critical for crops experiencing waterlogged conditions. Moreover, incorporating ecological principles such as increasing plant diversity by incorporating crop rotations and adaptive management via delayed planting dates and adjustments in nutrient management will be critical for fostering climate resilience in row-crop agriculture moving forward.

## Teaser

A review exploring biological, chemical, and physiological processes that occur between aboveground plant components and belowground (rhizosphere) networks that may be altered due to waterlogged conditions.

## Introduction

Above- and belowground linkages have long been identified as critical for numerous ecological processes in both natural and managed systems (Bardgett and Wardle, 2010; Wardle and Jonsson, 2014). The most fundamental example of a positive above- and belowground linkage comes in the form of net primary production outcomes, which are often driven by soil nutrients made available by microbial communities. These plant–soil–microbe interactions are central to plant growth and overall productivity in all terrestrial landscapes (Thakur, 2020). Many in the literature have also illustrated that aboveground community composition drives overall plant biomass allocation, microbial composition, and litter decomposition, thereby influencing major biogeochemical cycles including carbon and nitrogen (De Deyn *et al.*, 2008; Wardle *et al.*, 2012). In the last decade or so, work has shifted to focus more on how above- and belowground linkages are altered due to global climate change (Tylianakis *et al.*, 2008; Bardgett and van der Putten, 2014; C. Wang *et al.*, 2021). Both direct and indirect effects of climate change can have enormous impacts on both above- and belowground communities. In turn, this has the potential to alter how these ecosystem components interact, which could lead to trophic mismatches, community shifts, and leaky nutrient cycles ultimately influencing the type of ecosystem services that can be delivered in various systems.

Global change biology influences above- and belowground interactions, altering chemical, biological, and physiological processes and overall plant survival in natural landscapes. Climate stress can drastically alter biogeochemical cycles due to shifts in biomass allocation, whereby plants often allocate more resources belowground (Quan *et al.*, 2020). For instance, a greater amount of root exudates have been reported in plants experiencing and recovering from drought (de Vries *et al.*, 2019). It is hypothesized that releasing more exudates is a plant’s way of recruiting beneficial microbes needed during recovery (Sasse *et al.*, 2018; Williams and de Vries, 2020). From a biological perspective, disturbance has been widely shown to shift food webs, which in turn, can influence plant development and survival (Martin and Sprunger, 2022). Elderd (2006) found that belowground trophic interactions were altered in riparian zones during major flooding events in a manner that benefited plant performance. Flooded conditions increased the presence of wolf spiders, which then preyed on the herbivorous leafhoppers, reducing overall plant herbivory. This demonstrates a key finding where increased predation pressure led to overall greater plant performance. Numerous studies have demonstrated how climatic stress impacts plant physiology at multiple stages in a plant life cycle, altering above- and belowground dynamics (Bailey-Serres *et al.*, 2012; Kaur *et al.*, 2020; Oram *et al.*, 2020). Lastly, plant invasions within natural systems tend to increase under climate stress in both forest and grassland systems, impacting the overall survival of native plants (Bardgett and van der Putten, 2014).

**Fig. 1.**
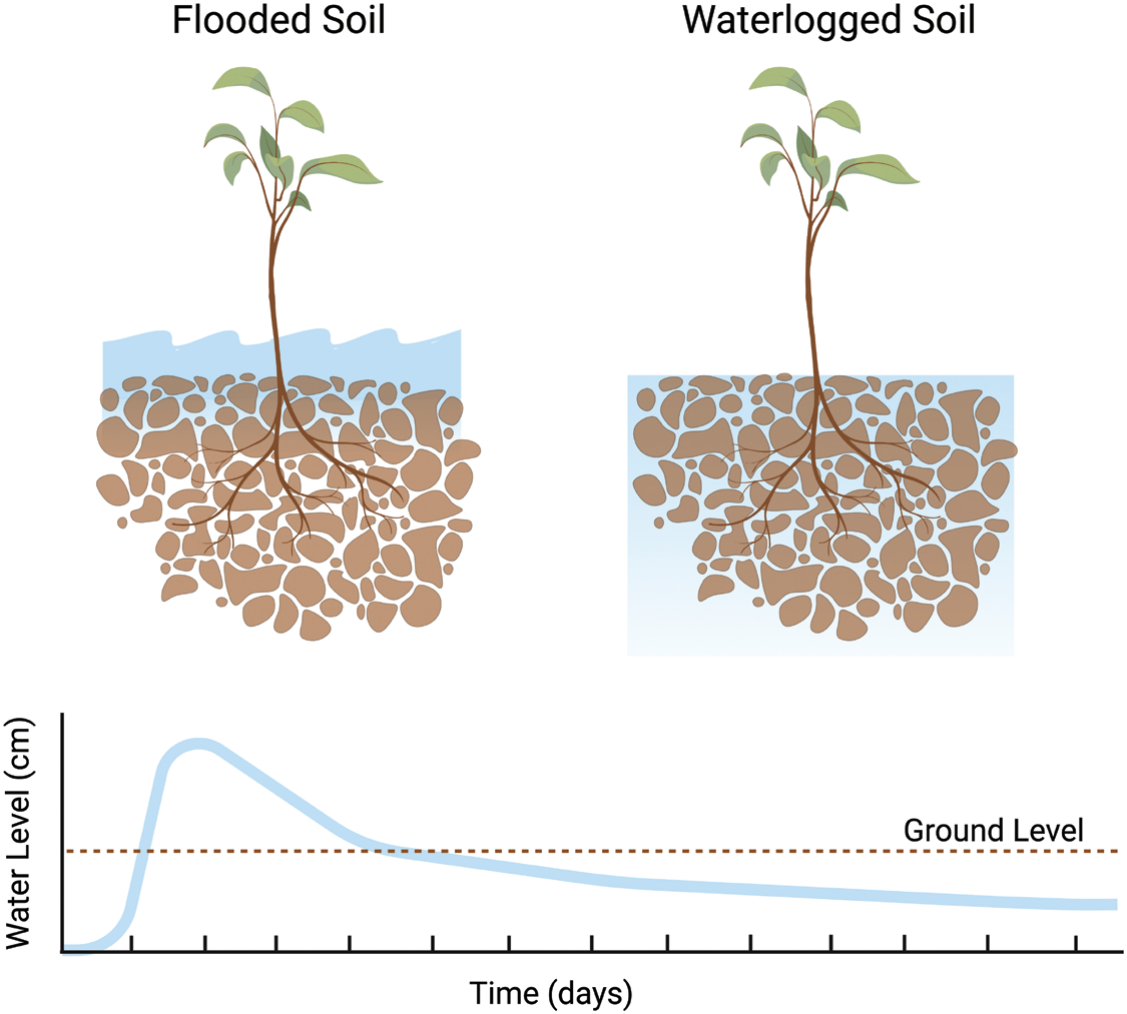
Conceptual figure of flooded versus waterlogged soils in agricultural landscapes. Flooded conditions are acute and generally dissipate within a few days of the initial flooding event. In contrast, waterlogged soils occur when soil pores are saturated with water, and such conditions can persist for several days following extreme rainfall events.

Extreme precipitation events will continue to increase due to anthropogenic climate change. In many regions of the world, intensive rainfall events are expected to increase, followed, by periods of intense drought (IPCC, 2022). These intense and variable rainfall events often lead to flooding and subsequent waterlogged conditions, which has proven to be a hazard that has consequences for plants, animals, and humans (Williamson and Wardle, 2007; Mallakpour and Villarini, 2015). According to Kaur *et al.* (2020) flooding is a condition in which all or part of the plant is submerged under water, while waterlogged conditions occur when soil pores are saturated with water. Flooding is often an acute event that generally lasts for 1–3 d, though subsequent waterlogging can persist for several days on end following an extreme rain event (Fig. 1). Both conditions lead to excess moisture and can significantly alter plant growth, and for this reason the two terms are often used interchangeably. Every year, flooding continues to impact an estimated 17 million km^2^ of land globally (Voesenek and Sasidharan, 2013). Heavy precipitation events in the USA have increased by up to 71% (Karl *et al.*, 2009). In particular, the Midwest has endured $7.7 billion losses due to flooding damage between 2013 and 2017 (Neri *et al.*, 2019). While it is clear that flooding events have adverse impacts on overall crop productivity, there is less understanding of how flooding and subsequent waterlogged conditions might impact above- and belowground linkages and key ecosystem functions, especially in row-crop agriculture.

In a recent review, Kaur *et al.* (2020) provided a detailed outline of how flooded conditions impact crop development and nitrogen losses in row-crop agriculture. However, they acknowledge that little is known regarding management strategies that can be implemented to foster adaptation and climate resilience in response to increased flooded conditions. Terrestrial ecosystems can adapt to global change biology in large part due to dynamics and interactions that occur between above- and belowground components of a given system. This review will explore biological, chemical, and physiological processes that occur between aboveground plant components and belowground (rhizosphere) networks that may be altered due to waterlogged conditions. The review will also place a special emphasis on crop responses to variable rainfall and the occurrence of flooding events and attempt to elucidate key mechanisms that are critical for climate resilience in Midwestern cropping systems (i.e. cropping systems that do not typically incorporate long flooding periods during cultivation as seen in rice). We argue that a stronger understanding of above- and belowground linkages that occur within natural and unmanaged landscapes could lead to climate adaptation and enhanced resilience within cropping systems. Given that above- and belowground linkages are key to ecosystem processes including productivity and nutrient cycling, further understanding the mechanisms that drive these interactions will be critical in developing management plans for farmers and landowners to foster greater climate resilience.

**Fig. 2.**
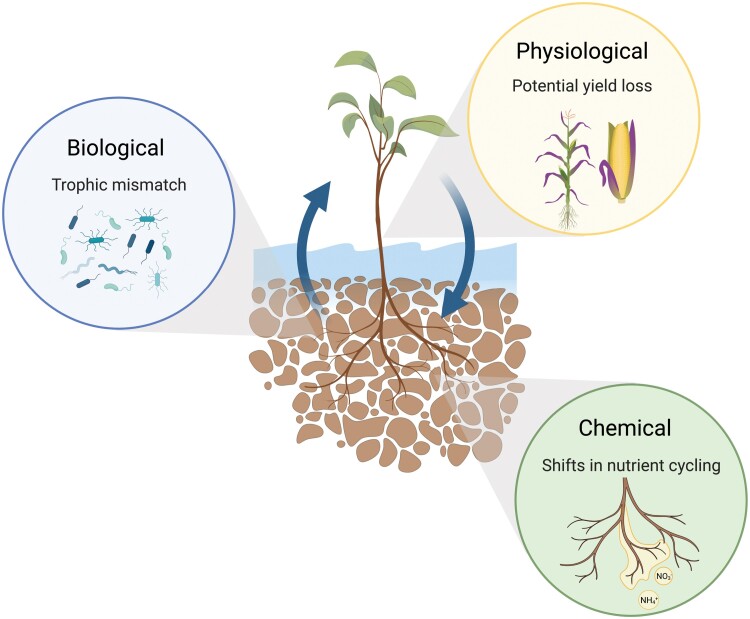
Overview of how above- and belowground processes can influence biological, chemical, and physiological mechanisms in flooded or waterlogged conditions.

Here we outline how key above- and belowground linkages are altered due to flooding or waterlogged conditions and how these interactions influence biological, chemical, and physiological processes (Fig. 2). For each process, we also highlight what lessons can be applied to agricultural systems to enhance overall cropping system resilience under excess moisture conditions.

## Biological processes: food web dynamics and trophic mismatches

Global change biology adversely impacts biological processes within unmanaged terrestrial landscapes. In terms of flooding, excess soil moisture can change the soil habitat drastically as it transitions from aerobic to anaerobic conditions (Visser and Voesenek, 2005). A reduction in oxygen inhibits a wide range of soil processes including mineralization, decomposition, and plant nutrient uptake because the microbial community, which is the foundation for these processes, is substantially altered (Schuur and Matson, 2001). Through decomposition and energy transfer, above- and belowground plant components have a significant impact on their respective food webs. Coined green and brown food webs by Thakur (2020), these food webs are interlinked through trophic interactions. However, Thakur (2020) posits that because green and brown food webs respond differently to climate change, it can be expected that trophic mismatches will likely occur. This could have adverse impacts on above- and belowground interactions and as a result, alter ecosystem processes (Table 1). For instance, green food webs might be more susceptible to global change biology, while brown food webs may be slower to react. These shifts in responses to climate change will lead to imbalances between green and brown food webs. Consequences of these imbalances could include reduced aboveground production, leading to a reduced food source for green food webs, which could shift carbon and nitrogen mineralization rates (Thakur, 2020). Reductions in nutrient cycling could drastically shift the brown food web, whereby decomposition pathways could be more bacterial dominated versus fungal dominated (Ferris *et al.*, 2001), altering the rate that decomposition occurs in a given system.

**Table 1.** The effect that flooding has on biological processes in managed and unmanaged terrestrial landscapes

Ecosystem	Above- and belowground linkages	Ecological process	Positive or negative effect on ecosystem service	Reference
Riparian zones	Plant functional diversity impact on Collembola assemblages	Flooding shifted plant species richness and functional diversity	Collembola responded to shifts in plant community but not to flooding	Abgrall *et al.* (2017)
Grassland	Plant diversity and soil food webs	Shifts in soil food web structure and function	Increased diversity did not shield soil food webs from flooding effects and soil food web complexity decreased with flooding	Wagner *et al.* (2015)
Abandoned agricultural field	Above- and belowground food web	Shifts in food web	Above- and belowground communities decreased due to flooding	Roeder *et al.* (2018)
Riparian zones	Above- and belowground food web	Shifts in food web	Predators only prevented herbivore damage with flooding	Elderd (2006)
Range of terrestrial ecosystems	Abiotic factors influence root defense investment, root signaling ability, herbivore interactions	Investment of plant resources in fewer roots, defense investment, root exudates	Increased root susceptibility to herbivore pressure, decreased ability of plant roots to signal enemies of root herbivores	Erb and Lu (2013)

Numerous groups of soil biota make up the soil food web, creating complex relationships across trophic levels that have varied responses to global change biology (Nielsen *et al.*, 2015). As a result, trophic mismatches can occur solely within ‘brown food webs’. Moreover, the impact that plant community composition has on various trophic groups can also change, demonstrating that different taxa may have contrasting responses to above–belowground dynamics. For instance, Wagner *et al.* (2015) explored how microbial and nematode communities responded to natural floods within riparian zones and whether plant diversity reduced the impact of flooding on the soil food web. The team found the flooding drastically reduced gram-negative bacteria, while fungi and nematode communities were less impacted. In fact, nematodes seemed to positively respond to increased plant biodiversity, which may have helped stabilize communities under flooding. That different trophic levels had altered responses to plant diversity under disturbance demonstrates the complex relationships that can occur between above- and belowground compartments and sheds light on the role that soil food webs can play in climate resilience (Catovsky *et al.*, 2002).

### Biological lessons for agriculture

There are numerous lessons that agriculture can take from natural based systems including how above- and belowground dynamics influence biological processes in the face of climate change. For instance, several studies demonstrate that plant-growth-promoting rhizobacteria are particularly susceptible to climatic stress, which has serious implications for crop productivity (Table 1; Tewari and Arora, 2013; Francioli *et al.*, 2021). There are several mechanisms by which plant-growth-promoting rhizobacteria can enhance plant growth including N fixation, the production of indolic compounds, siderophore production, enzyme activity, and phosphate solubilization (de Souza *et al.*, 2015). Anoxic conditions within a given agroecosystem created due to flooding or waterlogged conditions can drastically influence rhizobacteria and the key mechanisms that aid in plant growth promotion (Francioli *et al.*, 2021). For instance, de Souza *et al.* (2015) report that abiotic stress including flooding, leads to greater endogenous ethylene production within plants, which adversely impacts growth. The bacterial enzyme 1-aminocyclopropane-1-carboxylate deaminase can reduce ethylene production and many studies have demonstrated that production of this enzyme increases in the presence of plant-growth-promoting rhizobacteria and can ameliorate flooding stress (Grichko and Glick, 2001; Tewari and Arora, 2013; Barnawal *et al.*, 2012). Moreover, flooded conditions can even impact the quality and quantity of root exudates entering a system, which is the first step needed for a plant to recruit plant-growth-promoting bacteria (Smucker and Erickson, 1987; Vives-Peris *et al.*, 2020). Henry *et al.* (2007) report that flooded conditions increased the amount of total organic carbon present in root exudates by 45%. This exchange of resources and energy between above- and belowground components can alter overall plant productivity, even if belowground production is enhanced to recruit more beneficial bacteria during times of stress.

Similar to natural ecosystems, trophic mismatches have been documented within agricultural landscapes in response to global change manipulations. For example, Guyer *et al.* (2021) identified that root pests could be exacerbated under climatic change due to increased herbivory and reduced biological control agents. On the other hand, Erb and Lu (2013) note that flooding may decrease the abundance of root feeding herbivores as well as natural enemies. That said, certain larvae can adapt to flooded conditions threatening plants and creating a trophic mismatch, given that natural enemies have not adapted to the flooded conditions. As flooding events continue to intensify, trophic mismatches that lead to reduced biological control could become devastating for crop production in the future. Additionally, future research should be conducted on a wide range of crops, as different species will have different strategies to combat root herbivory under flooded conditions. Surprisingly, there is a lack of research regarding the impact that flooding has on soil food webs within agroecosystems, which is unfortunate given that soil food webs can serve as a major indicator of soil health (Wagner *et al.*, 2015; Martin and Sprunger, 2022). However, examples from unmanaged terrestrial landscapes demonstrate that microbes, nematode communities, and macroinvertebrates do tend to shift under flooded conditions, disrupting trophic interactions and soil food web health (Wagner *et al.*, 2015; González-Macé and Scheu, 2018; Francioli *et al.*, 2021). This in turn can have cascading effects on nitrogen and carbon mineralization, ultimately impacting plant nutrient uptake (Neher, 2001). Future agricultural trials should assess how soil flooding impacts overall soil food web dynamics, soil health, and crop productivity. Depending on how certain trophic levels respond to climate change, certain taxa could serve as key indicators of resilience or further stress in any given agroecosystem.

## Chemical processes: carbon and nitrogen balance

Exploring how plant biomass allocation impacts chemical processes such as carbon sequestration and nitrogen retention is perhaps the most commonly explored above- and belowground linkage to date (Bloom *et al.*, 1985; Jenkinson *et al.*, 1991; Cox *et al.*, 2000; Bardgett and Wardle, 2003). This has led researchers to explore how abiotic and biotic factors drive biomass allocations that could have implications for biogeochemical cycles on a global scale (Jackson *et al.*, 1996). For instance, in water-limited ecosystems, root growth is stimulated to deeper depths, impacting the carbon balance in arid systems (Schenk and Jackson, 2002). Over time it has become widely understood that greater biomass allocation towards root systems is a strong predictor of carbon sequestration in a wide range of ecosystems (Rasse *et al.*, 2005; Sprunger *et al.*, 2018). Thus, understanding the mechanistic drivers that control biomass allocation can provide insight into how above- and belowground dynamics aid in climate mitigation, especially in the face of global change biology (Bardgett and Wardle, 2010). For example, in a global meta-analysis, Terrer (2021) found that increased plant biomass under elevated CO_2_ led to a decrease in soil carbon, in contrast to what is found in normal conditions. This decrease in soil carbon occurred because plants with larger biomass led to greater mining of soil nutrients that outpaced the ability for roots to contribute to soil C accumulation.

Given that climatic conditions are altering plant–soil interactions that have negative impacts on the soil carbon balance, many researchers are working to assess if introducing plant biodiversity is effective at creating more resilient ecosystems. Shifts in plant community composition influence both biomass allocation and changes to the quality of carbon inputs via root exudates, which has large implications for soil carbon accumulation in both labile and more stable pools (Treseder *et al.*, 2005; Panchal *et al.*, 2022). There is also ample evidence that increased stand diversity in forests is associated with climate resilience (Silva Pedro *et al.*, 2015; Hisano *et al.*, 2018; Morin *et al.*, 2018). Polyculture systems tend to allocate more resources belowground due to plant complementarity. This ultimately results in greater nutrient use efficiency and resiliency (Silva Pedro *et al.*, 2015). This trait diversity can lead to greater carbon storage and nitrogen uptake as well, which helps with overall climate resilience (Chen *et al.*, 2018). Moreover, greater plant diversity is often, though not always, associated with more diverse soil biota, creating belowground networks that are better adapted to withstand climatic stress (De Deyn *et al.*, 2005; Chen *et al.*, 2019; Liu *et al.*, 2020).

### Chemical processes and lessons for agriculture

The same ecological theories surrounding biodiversity and biochemical processes can be implemented within agroecosystems and have profound impacts on climate resilience. For example, Bowles *et al.* (2020) demonstrate that crop diversification is critical for enhanced yield stability over time, even in times of climate stress such as drought. The mechanisms that can explain greater crop yield stability can be attributed to above- and belowground linkages occurring in more diverse cropping systems (Seipel *et al.*, 2019). For example, greater soil organic matter storage due to a greater quantity and quality of above- and belowground residues entering a system often can foster enhanced moisture retention in drought years (Rawls *et al.*, 2003; Zhang *et al.*, 2021). This diversity of inputs is similar to plant complementarity that occurs in natural systems, whereby legumes are providing additional nitrogen credits via N fixation (Kahmen *et al.*, 2006; Cardinale *et al.*, 2007). Similar to unmanaged landscapes, more diverse crop rotations increase the soil microbial community and enhance overall soil health, which can aid in disease suppression and overall climate resilience (Tiemann *et al.*, 2015; Peralta *et al.*, 2018; Sprunger *et al.*, 2020).

Recent research suggests that flooding will lead to substantial losses of nitrogen and phosphorous in agroecosystems. Nitrogen and phosphorous loading into streams increases during large flooding events (Verma *et al.*, 2018). Additionally, nitrous oxide emissions from agricultural landscapes peak in flooded conditions (Hansen *et al.*, 2014). However, little is known regarding how flooding may impact crop biomass allocation and nutrient cycling. That said, there is reason to believe that flooded conditions could stimulate fine root production as roots continue to forage for nutrients deeper in the soil profile (Dill *et al.*, 2020), given enhanced nitrate leaching. While enhanced root production may have a positive effect on belowground soil C dynamics, the trade-off will likely be reduced aboveground production. While numerous studies have been conducted on drought stress and soil carbon dynamics in agroecosystems (Zhou *et al.*, 2016; de Vries *et al.*, 2019), more research is needed to better understand the carbon balance of row-crop agriculture under flooded conditions. Table 2 highlights the limited number of studies that have explored above- and belowground dynamics in the context of flooding and biogeochemical processes.

**Table 2.** The effect that flooding has on chemical processes in managed and unmanaged terrestrial landscapes

Ecosystem	Above- and belowground linkages	Ecological process	Positive or negative effect on ecosystem service	Reference
Row-crop agriculture	Crop growth and root-associated rhizobacteria	Root exudation, nutrient transfer, growth stimulation, and stress tolerance	Rhizobacteria enhanced lateral root growth in ­response to variable rainfall	Czarnes *et al.* (2020)
Row-crop agriculture	Crop growth and root-associated rhizobacteria	Root exudation, stress tolerance	ACC deaminase-containing rhizobacteria alleviate heavy metal accumulation, increase root growth and crop establishment	Tewari and Arora (2013)
Swamp forest	Flooded conditions limits decomposition processes	Nutrient cycling	Greater presence of belowground biomass under flooded conditions led to reduced nitrogen losses, as N and P accumulate in the form of microbial biomass	Kemp *et al.* (1985)

ACC: 1-aminocyclopropane-1-carboxylate.

## Physiological processes

Plant communities develop strategies to secure resources when under abiotic and/or biotic stress (Wright *et al.*, 2004; Erb and Lu, 2013; Fort *et al.*, 2016). Certain communities will invest photosynthate into leaves (i.e. the leaf economic spectrum coined by Wright *et al.*, 2004), while other communities may invest more resources belowground to root traits (Bloom *et al.*, 1985; Fort *et al.*, 2016). These physiological trade-offs are foundational to above- and belowground linkages, as resources are being traded between various plant compartments and certain traits are prioritized based on the community’s survival strategy (Table 3). Oram *et al.* (2020) found that in an intensively flooded grassland, plant communities characterized by low specific leaf area, low leaf nitrogen, and high leaf content were better able to resist and recover from flooding events. This is likely due to the fact that these slower growing grasslands are more conservative with resources compared with fast-growing communities and are able to maintain biomass even under flooded conditions. In areas where prairies are being restored, land managers should be strategic in thinking about plant survival strategies when seeding a given field.

**Table 3.** The effect that flooding has on physiological processes in managed and unmanaged terrestrial landscapes

Ecosystem	Above- and belowground linkages	Ecological process	Positive or negative effect on ecosystem ­service	Reference
Riparian zones	Biological invasion leading to excess weed pressure Roots of weeds compete for soil nutrients.	Competition for nutrients leads to nutrient deficiencies	Reduced net primary productivity Crops more susceptible to pest and disease	Wei *et al.* (2015); Sun *et al.* (2022)
Marsh	Above- and belowground plant production	Net primary productivity and carbon accumulation	Species in historically stable marshes had reduced above- and belowground biomass while deteriorating marshes were more tolerant of flooding	Kirwan and Guntenspergen (2010)
Grazed wetland	Above- and belowground traits and biomass allocation	Plant growth, vigor, and Net primary productivity	Root dry matter and root tissue density decreased	Purcell *et al.* (2019)

Flooding can also have detrimental impacts on plant roots in a wide range of ecosystems. Anoxic conditions lead to lower root respiration and inhibition of root growth, reducing the ability for plant roots to forage for additional nutrients (Sairam *et al.*, 2008). In grassland systems, root biomass has been shown to decrease under flooded conditions (Oram *et al.*, 2020). Reductions in root growth due to flooding can also impact symbiotic relationships that might occur between plants and microbes, further inhibiting overall plant development. That said, there is evidence that species-rich communities are less impacted by flooding relative to monocultures (Wright *et al.*, 2017). Mechanisms that explain this include greater specific leaf area, plant height, and root aerenchyma, all of which promote higher amounts of gas exchanges. Thus, even under climatic stress, the more diverse grasslands were able to continue to grow leading to more ecosystem stability relative to monoculture systems.

### Physiological lessons for agriculture

Part of the crop response to flooding is driven by low-oxygen levels or high carbon dioxide levels in the soil. Crops experiencing flooding will reduce photosynthetic and respiration rates due to stomatal closure (Kozlowski, 1984; Oosterhuis *et al.*, 1990). Additionally, chloroplasts and cellular membranes in leaf tissue begin to degrade after 3 d of flooding (Ren *et al.*, 2016). Flooding can induce stress symptoms in plants such as leaf chlorosis, wilting, and stunting, in addition to more severe symptoms like necrosis and plant death (Fausey *et al.*, 1985; Boru *et al.*, 2003). Delays in early-season growth may reduce yield potential, early-season nutrient uptake, and vegetative biomass production/leaf area formation (Mukhtar *et al.*, 1990; Caudle and Maricle, 2012). Similar to natural systems, crops have their own survival strategies that may influence how a certain crop may respond to climate stress. Reduced leaf area and height can decrease primary productivity by reducing the ability of the plant to intercept light, which could limit grain yield (Table 3). Photosynthetic ability could also be influenced by the reduction or change in leaf pigment ratios associated with leaf chlorosis. Smaller plants could also increase the likelihood of surface runoff caused by intense storm events, so identifying crop cultivars and hybrids with both improved rooting ability and shoot biomass production is key to help reduce the negative environmental effects of early-season flood events.

Additionally, elevated levels of soil water reduce the oxygen available to the root systems dramatically, resulting in a buildup of carbon dioxide that may be more detrimental to plant survival (Boru *et al.*, 2003). Anoxic conditions in the soil may stimulate adventitious root development (Wenkert *et al.*, 1981) as well as root cortical aerenchyma (Armstrong *et al.*, 1994; Dill *et al.*, 2020). These responses all have the potential to change how crops absorb nutrients from the soil solution and the way in which plants utilize absorbed nutrients, and may affect source–sink relationships and limit crop yield production. Under periods of flooding less than 10 d in duration, corn has been shown to produce adventitious roots as well as aerenchyma, which consists of air-filled cavities that allow for gas diffusion from a non-flooded area to the flooded cells (Zaidi *et al.*, 2004). Waterlogging-tolerant corn lines also produced more crown roots under waterlogging compared with less-tolerant lines (Zhai *et al.*, 2013). Corn roots form aerenchyma within 24 h of flooding through programmed cell death of cortical cells (Bailey-Serres *et al.*, 2012). However, creating these cell types may influence nutrient uptake as well as susceptibility to soil-borne pathogens. Application of N prior to flooding corn did not affect grain yield (Kaur *et al.*, 2017; Dill *et al.*, 2020), though height was improved post-flooding in plots that received a pre-plant N application compared with those that did not receive the pre-plant N application (Dill *et al.*, 2020). Altering the pathway for nutrient movement through cortical cells to the vascular bundle may delay nutrient uptake and transport to shoot tissue. Application of N post-flood has improved corn yield (Kaur *et al.*, 2017; Ren *et al.*, 2016; Dill *et al.*, 2020) and brassica yield (Zhou *et al.*, 1997), though studies to date have not conducted a fertilizer rate response to better understand the degree of uptake and utilization for grain yield production post-flood.

Flooding can also reduce nitrogen fixation in soybeans by limiting associations with rhizobacteria (Sallam and Scott, 1987) and suppressing nitrogenase activity in the roots (Sprent, 1969). Rhizobial associations typically begin at vegetative stage 2 (staging method as described in Fehr *et al.*, 1977), and excessive water may limit the formation of nodules leading to reduced yield or grain quality at the end of the season (Henshaw *et al.*, 2007). Application of N post-flooding in the preceding corn crop has been shown to increase soybean yield in some cases (Kaur *et al.*, 2017), suggesting management of N in the previous year in a field prone to flooding may affect future crops. Additionally, flooding can result in the formation of aerenchyma in soybean roots by triggering cell division and creating a cell layer between the cortex and epidermis after 2–4 d (Rhine *et al.*, 2010; Shimamura *et al.*, 2010). A reduced ability of root tissue to absorb nutrients from the soil could also lead to increased nitrogen loss due to excessive precipitation. Some species, such as wheat and barley, also induce the formation of radial oxygen loss barriers that prevent the loss of oxygen being transported from shoots to root tips (Jia *et al.*, 2021). Waterlogging may also affect the production of root exudates in crested wheatgrass, which could affect nutrient uptake and microbial associations (Henry *et al.*, 2007).

## Species invasion

It has been widely documented that with global change biology there is an increase in invasive alien plants within unmanaged terrestrial systems, which has a sweeping impact on physiological, chemical, and biological processes, altering key above- and belowground linkages (Turbelin and Catford, 2021). Invasive species are often able to thrive in ecosystems that have been disturbed by an extreme climatic event, exploiting areas once dominated by native species that are unable to adapt to new conditions (Thuiller *et al.*, 2008; Catford *et al.*, 2019). Once an invasive species moves into a given space, their presence can directly start to change key ecosystem processes. For example, invasive species can alter above- and belowground litter within a given system, ultimately changing decomposition rates and nutrient cycling (Liao *et al.*, 2008; Kurokawa *et al.*, 2010). Invasive species can also alter plant–microbe interactions that impact nutrient availability in a given system. Sun *et al.* (2022) demonstrated that an invasive species (*Sphagneticola trilobata*) had a greater rate of mycorrhizal colonization, greater alkaline phosphomonoesterase-producing bacteria, and greater overall abundance of bacterivorous nematodes relative to the native species in a mixed polyculture community. This demonstrates that invasives are able to exploit available nutrients by fostering bacteria–nematode interactions more efficiently than their counterpart native species. As climatic extremes such as flooding and drought increase, exploitation of resources and alteration of chemical and biological processes by invasive species will only continue (Turbelin and Catford, 2021). Species invasion leads to overall losses in biodiversity, which in turn leads to reduced ecosystem function (McGeoch *et al.*, 2010).

### Lessons for agriculture: weeds

Agricultural systems are under constant invasion from weed species. Thus, understanding the associated above- and belowground dynamics that occur during species invasion in unmanaged terrestrial landscapes could shed light on weed pressures within agricultural systems. Waterlogging or flooding may lead to changes in the weed populations in fields in addition to affecting crop growth and development. Annual variation in temperature and soil moisture status can affect germinating weed populations. In some systems such as rice production, weed suppression through flooding is a key management strategy (Ismail *et al.*, 2012). Flooding rice fields in October after rice harvest but prior to soybean planting in March reduced weed presence by 43–99%, but subsequent yields of soybeans were reduced by 19–25% compared with non-flooded controls (Koger *et al.*, 2013). In the case of extreme volume and frequency of precipitation, as was observed in the US Midwest in 2019, weed emergence of species like giant foxtail and giant ragweed was delayed or was not observed compared with the drier seasons of 2020 and 2021 (Essman, 2022). Anecdotal information is available in some areas on how species shifts occur as a result of wet conditions, though published work from agricultural fields is severely lacking.

Researchers observed reductions in biomass and changes to the root:shoot ratio in *Elytrigia repens*, *E. intermedia*, and their hybrid (all perennial grasses), though the degree of the flooding effect was greater for some taxa than others suggesting variability in their tolerance levels (Mahelka, 2006). A wild ancestor of wheat, *Aegilops tauschii*, was more tolerant than wheat to waterlogged conditions and saw gains in its competitiveness under flooding (whereas wheat competitiveness decreased) (N. Wang *et al.*, 2021). Depending on the source of the waterlogging (slow infiltration versus from a water body), novel species may be introduced to fields as was observed with *Nicotiana glauca* in Australia (Florentine *et al.*, 2006). Distribution within fields of seeds may also be influenced by precipitation flow within the field, as the rapid spread of surface-spread seed in a cotton field was attributed to heavy precipitation (Norsworthy *et al.*, 2014).

Similar to natural systems, where invasive species expand under flooded conditions, excess water conditions will also impact a farmer’s ability to manage weeds. Pre-emergent herbicides are effective at controlling many agriculturally relevant species, and often require some precipitation for activation (1–2 cm precipitation). Successful weed control in corn was determined to need 5–10 cm precipitation within 15 d of application to effectively incorporate herbicides and facilitate ­uptake by weeds (Landau *et al.*, 2021). However, rainfall totals greater than those assessed by Landau *et al.* (2021) may result in challenges with retaining applied herbicides (Ramesh *et al.*, 2017) or applying them at all (field conditions too wet to facilitate a timely application). Poor weed control may impact a crop’s ability to grow and produce yield, and may affect agricultural ecosystems. That said, even weeds, while viewed as unfavorable in cropping systems, can serve as nutrient cycling materials in agricultural systems (Lindsey *et al.*, 2013). Nutrient release from weed residue decomposition tends to stabilize after 4–6 weeks (Lindsey *et al.*, 2013; Harre *et al.*, 2014).

An ecological approach that agriculture has adopted to enhance nitrogen release has been to increase biodiversity through the use of cover crops. For instance, aboveground and belowground N release from leguminous cover crops also has been shown to stabilize after 4–6 weeks (Jani *et al.*, 2015; Sievers and Cook, 2018). Mineralization of cereal rye has been shown to extend well beyond 6 weeks, with more N being released from belowground tissue than aboveground tissue (Sievers and Cook, 2018; Singh *et al.*, 2020). That said, these added nutrients from cover crops could be lost under waterlogged conditions. Moisture during mineralization was closely controlled in laboratory assays to ensure <60% water-filled pore space was achieved (Lindsey *et al.*, 2013; Jani *et al.*, 2015) and field assessments reported 40% volumetric water content or below during mineralization assays (Sievers and Cook, 2018; Singh *et al.*, 2020). In the event of water levels rising beyond these levels, it is possible that N released during mineralization could be lost to denitrification or leaching, which would affect its contribution to the soil nutrient pools.

## Managing for climate resilience within agriculture

### Cover crops

**Fig. 3.**
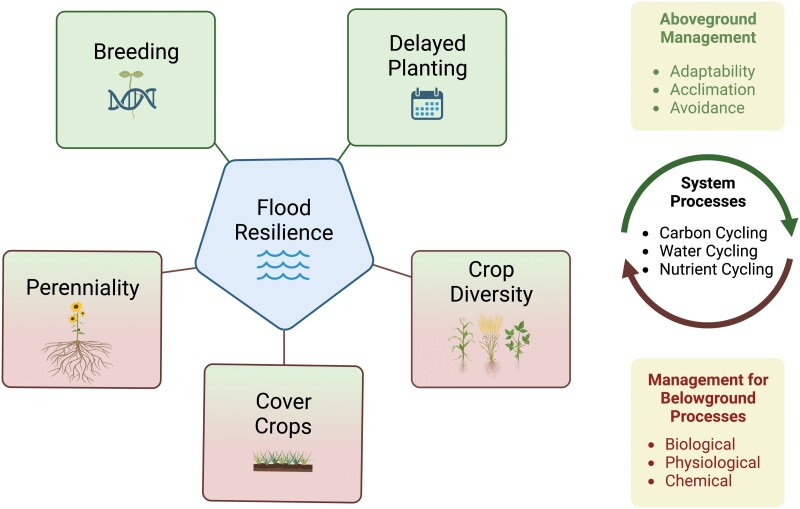
Potential management strategies that could be implemented to enhance flood resiliency and aid with overall climate adaptation within row crops.

There are a suite of farming management decisions and a variety of scientific innovations that could be used to enhance climate resilience within agriculture (Fig. 3). For example, the use of cover crops has been cited as a potential management practice to help overcome waterlogged or flooded conditions through improving soil structure and improving water infiltration (Haruna *et al.*, 2020; Kaur *et al.* 2020). Much of the benefit is long-term, and successful establishment of cover crops each year is key to ensuring that benefits are realized. Increasing plant cover of soil has been shown to improve surface water retention and reduce surface runoff (Durán Zuazo and Rodríguez Pleguezuelo, 2008). Reducing water movement rates across the surface at the field scale may increase the likelihood of water infiltration and decrease the volume of water flowing to low-lying areas in fields. Additionally, cover crops may prevent the formation of soil crusts due to flooded conditions as may occur in clean-tilled conditions by reducing soil surface strength (Folorunso *et al.*, 1992; Liu *et al.*, 2019; Griffiths *et al.*, 2021). This may impact the duration of flooding in a field, as well as the potential for subsequent flooding episodes. Rye, vetch, and wheat have all been reported to increase the saturated hydraulic conductivity in some studies (Haruna *et al.*, 2020), though not every group observed an increase. Multiple cover crops have been reported to increase the water infiltration capacity (likely through greater macropore presence) by anywhere from 11 to 629% (Haruna *et al.*, 2020).

While some researchers have observed reductions in soil moisture with cover crops prior to planting corn (Ewing *et al.*, 1991), long-term use of rye cover crops in Iowa (13 years) resulted in increased soil water storage by 21–22% in the upper 30 cm (Basche *et al.* 2016), though most other studies reported smaller increases in available water content and field capacity (Haruna *et al.*, 2020). Because water level in the soil prior to flooding is a major predictor of flooding events (Neri *et al.*, 2019), it is possible this could improve crop resilience by reducing the duration of flooding in crop fields. Moreover, given that flooded conditions will likely lead to greater nitrogen loss, incorporating cover crops may be critical for balancing the nitrogen cycling in flooded conditions as grass cover crops are especially effective at reducing nitrate leaching (Thapa *et al.*, 2018).

Cover crops also have weed suppressive capabilities, which could affect the plant species diversity and yield potential within a production system (Flood and Entz, 2019; Haramoto, 2019; Essman *et al.*, 2020). For example, grasses and mixed cover crops substantially suppress weeds, while legume cover crops are less effective (Baraibar *et al.*, 2018). Weed suppression from cover crops is also essential, as weed pressures will likely increase under flooded conditions as mentioned above. Moreover, the ability of cover crops to suppress weeds demonstrates another key above- and belowground linkage that fosters overall ecosystem resilience. Cover crop residue retained on the surface impedes seed germination and suppresses overall weed growth. Living roots of the cover crop may also aid in weed suppression early on in the growing season. More research at the intersection of cover crops and climate resilience is needed to understand the full potential of different cover crops in climate adaptation.

### Planting date

One management practice that may enable producers to better manage environmental stress is planting date. Earlier planting of summer annual crops in the Midwest has historically been associated with greater grain yield, but rain events may limit field activities in the spring, effectively delaying planting date beyond the optimal window. Delayed planting may require the use of a variety or hybrid with a shorter relative maturity to ensure physiological maturity is achieved before the first killing frost, though a yield penalty may be incurred compared with planting a variety with a longer relative maturity (Assefa *et al.*, 2016; Sciarresi *et al.*, 2020). Another aspect associated with planting date is daylength differences that could influence endurance to flooding. Soybeans experiencing flooding during the reproductive stages experience greater yield loss than those flooded during vegetative stages (Scott *et al.*, 1989); delayed planting may help minimize yield losses from flooding by shifting the flood occurrence to vegetative stages. Many anecdotal sources state that survival during flooding will decrease with increasing temperature (Nielsen, 2015), but most publications examining flooding do not incorporate a planting date effect to observe the genotype × environment interaction for flooding response (Fausey and McDonald, 1985; Nelson *et al.* 2011).

### Crop diversification and perennialization

Agriculture is dominated by annual cropping systems, which likely make agricultural landscapes more susceptible to flooding relative to systems dominated by perennial landscapes. In 2019, the Midwest had the largest unplanted area, due to excess waterlogged conditions in which farmers were unable to get into their fields (Lawal *et al.*, 2021). Given that early summer floods are projected to increase in the Midwest, farmers should consider incorporating into their operations more perennial grasses or legumes, which can be grown for forage. Or if farmers want to focus on annual row crops, winter wheat (*Triticum aestivum)* or winter barley (*Hordeum vulgare*) should be considered. These crops will all be in the ground with living roots during the winter months prior to any extreme precipitation that might impact the growing season. Small-seeded brassica species such as camelina, carinata, and most recently oilseed pennycress may also have utility as a winter annual option. Inclusion of these species into rotations with altered growth habits, nutritional content, and low C:N components may further influence above- and belowground cycling dynamics. Extending crop rotations and including three or more species often enhances soil C accumulation (McDaniel *et al.*, 2014), which could lead to greater overall resilience to climate relative to monoculture systems or even a typical corn–soybean rotation. Flooded conditions will also exacerbate nitrate leaching, and a solution could be to lengthen crop rotations with alfalfa as a way to reduce N losses (Dinnes *et al.*, 2002). Additional aboveground residues, greater root production, and enhanced root exudation found within more diversified crop rotations serve as nutrient catchments and greatly contribute to reduced nitrate leaching (Malpassi *et al.*, 2000). These same mechanisms are likely critical for alleviating flooding and/or waterlogged conditions (Kaur *et al.*, 2020).

While not on the market yet, perennial grain crops are also being developed at the Land Institute (Salina, KS, USA). The goal of perennial grain production is to develop a crop that can compete with annual crops but delivers ecosystem services like perennials found in nature due to deep and extensive root systems. For example, intermediate wheatgrass (*Thinopyrum intermedium*) has been developed and marketed as Perennial Kernza^®^. Kernza has been shown to drastically reduce nitrate leaching and improve soil health (Culman *et al.*, 2013; Sprunger *et al.*, 2019). Perennial grain crops could be used as a climate adaptation tool in the future given farmers would need to just plant once and then harvest, with the crop re-growing year after year. Others suggest converting annual corn to flood-tolerant perennial bioenergy crops as a way to adapt to continued waterlogged conditions (Quinn *et al.*, 2015).

### Breeding

While introducing a perennial grain crop into a traditional row-crop operation may seem extreme to farmers, one method growers can use to adapt to flooded conditions is to grow cultivars or hybrids that are more tolerant of excessive water conditions. Flood tolerance can be defined as the minimal loss of yield after a flooding event compared with a non-flooded environment (Rosielle and Hamblin, 1981), or as the ability to produce high yield after flooding (VanToai *et al.*, 1994). The duration of the flood event as well as crop stage can influence the severity of symptom response (Fausey *et al.*, 1985; Scott *et al.*, 1989; Oosterhuis *et al.*, 1990; Ren *et al.*, 2014). In corn, longer periods of flooding resulted in decreased emergence and survival (Fausey *et al.*, 1985). Mukhtar *et al.* (1990) demonstrated that early-season flooding is the most detrimental to corn yield after a 10 d flooding period. Damage in corn was more evident when flooding occurred at early developmental stages, both in photosynthetic ability (Tian *et al.*, 2019) and grain yield (Ritter and Beer, 1969; Ren *et al.*, 2014). Early-season flooding in corn can severely limit yield potential by restricting early flower development that occurs between the V6 and V16 growth stages (Stevens *et al.*, 1986; Abendroth *et al.*, 2011). Stress during ear initiation and development could reduce ear size, kernel rows per ear, and also potential kernels per row. Silk development may also be negatively impacted, which could lead to poor pollination (Cárcova *et al.*, 2003). In soybean, early-season flooding (V2-7) is typically less detrimental to yield as compared with flooding during early to mid reproductive stages R1 to R5 (Scott *et al.*, 1989; Linkemer *et al*, 1998; Rhine *et al.*, 2010).

Researchers have made significant progress in breeding more flood-tolerant corn and soybean (Wu *et al.*, 2017; de Oliveira, 2021), but efforts for winter wheat and barley are just beginning (Mustroph, 2018). It is important to note that even in breeding, exploring key above- and belowground linkages is critical in assessing flood tolerance. For instance, quantitative trait locus trials have informed breeders to focus on key plant traits including aerenchyma formation, reducing radial oxygen loss, and root growth (Mustroph, 2018). Breeders have looked to rice as a study system to assess which traits seem to aid in the flood tolerance of rice crops. Ismail *et al.* (2012) found that the majority of plants that were flood tolerant were able to adapt to oxygen deficiency by having high amylase and pyruvate decarboxylase activity and fast coleoptile growth. A combination of variety trials and genome-wide selections should guide future breeding efforts in the quest to find flood-tolerant cultivars.

## Conclusions

Above- and belowground interactions have long been associated with important ecosystem services in unmanaged landscapes. However, these key interactions are threatened by climatic extremes that result in flooding, ultimately altering biological, chemical, and physiological processes in both managed and unmanaged landscapes. It is understood that climate change is driving instability in green and brown food webs (as well as within webs), though it is unclear ultimately how or when these webs will begin to stabilize and what the ultimate influence will be on the ecosystem. Species shifts in weed populations are anticipated, and existing methods for control may be less effective under extreme weather conditions. Adaptive management in row-crop agriculture, including increased biodiversity and perenniality that bolsters trophic interactions and nutrient balance, will be critical within flooded agroecosystems to mitigate the level of destabilization that is anticipated. Furthermore, we have demonstrated major areas in research especially in regard to rhizosphere processes, including plant–microbe interactions that may foster climate resilience within cropping systems. More research addressing both above- and belowground responses to flooded conditions and how these influences crop productivity and ecosystem function within agroecosystems is needed. Lastly, breeders should consider above- and belowground linkages when identifying key traits for flood-tolerant cultivars.
